# A genomic resource for medicinal plant research: chloroplast genome assembly and annotation of *Helicteres isora* L.

**DOI:** 10.3389/fpls.2026.1792650

**Published:** 2026-05-19

**Authors:** Sudarmono Sudarmono, Aditya Nugroho, Ratna Uli Damayanti, Aris Sudomo, Intani Quarta Lailaty, Surya Diantina, Yosephin Martha Maria Anita Nugraheni, Kartika Dyah Palupi, Diana Prameswari

**Affiliations:** 1 Research Center for Biosystematics and Evolution, National Research and Innovation Agency (BRIN), Bogor, West Java, Indonesia; 2 Research Center for Applied Botany, National Research and Innovation Agency (BRIN), Bogor, West Java, Indonesia; 3 Research Center for Ecology, National Research and Innovation Agency (BRIN), Bogor, West Java, Indonesia; 4 Research Center for Pharmaceutical Ingredients and Traditional Medicine, National Research and Innovation Agency (BRIN), Bogor, West Java, Indonesia

**Keywords:** chloroplast, genome, *Helicteres isora*, Helicteroideae, Indonesia

## Introduction

Rapid advances in plant genomics have opened up tremendous opportunities for understanding the genetic basis, evolution, and adaptation of various plant species, including medicinal plants. Genomic data is now an important foundation for studies in systematics, phylogenetics, conservation, and the sustainable use of biological resources. Medicinal plants have the potential to be developed as new model systems (emerging models) because they have a unique genomic architecture and regulatory pathways that are important for understanding secondary metabolism and adaptive evolution (Ray et al, 2022). The shift from laboratory model systems to non-model species in natural environments reflects the need to understand natural variation and molecular mechanisms in a more realistic ecological context. (Richards et al, 2017).

The chloroplast genome is an important component in the study of diversity, evolution, and the application of plant genetic techniques. (Daniell et al, 2016). In general, the chloroplast genome has a quadripartite structure consisting of large single copy (LSC), small single copy (SSC), and two inverted repeats (IR) regions, with a relatively conserved gene composition. Recent studies show that chloroplast DNA (cpDNA) is not only circular, but can also exist as a branched linear structure, reflecting a more dynamic and complex chloroplast genetic system than previously understood (Dobrogojski et al, 2020). A deep understanding of the chloroplast genome has fundamental and applied value, especially in plant biotechnology, although chloroplast genome modification still faces technical and biological challenges. (Olejniczak et al, 2016).

In the context of plant identification and authentication, chloroplast genomes play an important role in the development of DNA barcoding, especially for medicinal plants. However, the absence of a universal barcode locus for all plant groups makes the selection of informative chloroplast markers a crucial aspect in phytomedicine authentication and biodiversity conservation (Alam et al, 2024). Information obtained from chloroplast genomes, such as gene organization, codon usage patterns, and sequence variation, has been widely used for high-resolution phylogenetic analysis, molecular marker development, and comparative studies between species. In the context of medicinal plants, the availability of complete chloroplast genomes is becoming increasingly important as it can support species authentication, tracking evolutionary relationships, and exploring genetic diversity relevant to conservation and pharmacological utilization.

Screw tree (*Helicteres isora*), known locally as kayu ules, is one of the medicinal plant species that grows naturally and is still rarely cultivated. According to the 2024 IUCN Red List, this species is categorized as Least Concern. Morphologically, *H. isora* belongs to the Malvaceae family and is an upright shrub with a height of about 1–2.5 m, with red flowers (Kanthale and Biradar, 2017). The fruit is arranged in a spiral or twisted pattern, which is the basis for its common name, screw tree. (Kumbhani et al, 2017). Various parts of this plant, including the bark, roots, leaves, seeds, and fruit, have been reported to have medicinal properties, including for treating diarrhea (bark), diabetes, weakness in newborns, and ear infections (fruit), dysentery (seeds), and scabies (leaves). These pharmacological activities are related to the content of important bioactive compounds, such as betulinic acid, β-tocopherol, coumarin, β-sitosterol, saponins, flavonoids, tannins, and alkaloids (Dayal et al, 2015; Kanthale and Biradar, 2017; Meena et al, 2018).

Despite its high pharmacological value, *Helicteres isora* does not yet have a publicly available annotated chloroplast genome resource. Currently, only one chloroplast genome sequence from a closely related species, *Helicteres hirsuta* (Nguyen et al, 2024), has already been reported and provides an important comparative reference for genome annotation and structural characterization in this genus. The availability of plastome data from related taxa highlights the value of comparative chloroplast genomics for improving genome annotation accuracy and for supporting evolutionary and phylogenetic studies within Helicteres. However, the lack of comprehensive genomic information has limited evolutionary and ecological studies, particularly in understanding natural variation and local adaptation on a broader genomic scale (Song & Mitchell-Olds, 2011). Therefore, this study aimed to assemble, annotate, and characterize the complete chloroplast genome of *H. isora L*. as a basic genomic resource for medicinal plant research. This study focused on the analysis of plastome architecture, gene composition, and genome organization. The resulting chloroplast genome data are expected to serve as an important reference for future studies in systematics, evolutionary biology, genetic conservation, DNA barcoding, and bioprospecting, as well as a basis for formulating more effective conservation and management strategies for this valuable medicinal plant.

## Methods

### Plant materials

Fresh leaves were collected from a single individual of *H. isora* collected in Bosen village, North Mollo Sub District, South Timor Tengah District, East Nusa Tenggara Province, Indonesia (10°09’34.95” S, 123°35’03.32” E).

### DNA extraction

Fresh leaf tissue of *Helicteres isora* was used for genomic DNA extraction. Total genomic DNA was isolated using the SEPa Plant DNA Isolation Reagent Kit (KIT-9101) following the manufacturer’s instructions. The concentration and purity of the extracted DNA were initially assessed using a NanoDrop 2000 spectrophotometer (Thermo Scientific) by measuring the A260/280 and A260/230 ratios. DNA integrity was evaluated by 1% agarose gel electrophoresis. Accurate DNA quantification was performed using the Qubit dsDNA High Sensitivity Assay Kit (Thermo Scientific). Further assessment of DNA integrity was carried out using the Agilent 4150 TapeStation.

### Library preparation and sequencing

High-quality genomic DNA was used as input for library preparation using the xGen DNA Library Prep EZ UNI Kit (Integrated DNA Technologies, IDT). Genomic DNA was enzymatically fragmented to obtain the appropriate insert size, followed by ligation with Illumina-compatible adapters. PCR amplification was subsequently performed to generate full-length adapters containing unique index sequences for each library. The quality and quantity of the prepared libraries were evaluated using a Qubit Fluorometer, Agilent TapeStation, and quantitative PCR (qPCR). Paired-end sequencing (2 × 150 bp) was conducted on the Illumina NextSeq 2000 platform by PT. Genetika Science Indonesia. Base calling, demultiplexing, and FASTQ file generation were performed using Illumina DRAGEN BCL Convert v4.2.7.

### Chloroplast genome assembly and annotation

Raw reads were evaluated for quality using FASTQC v0.11.8 (Andrews et al, 2010). Adapter sequences, low-quality bases (quality score <30), nucleotide bias at the 5′ and 3′ ends, and potential contaminant sequences were removed using Trimmomatic v0.39. The trimming process employed the following parameters: TruSeq3-PE.fa:2:30:10, SLIDINGWINDOW:4:28, LEADING:28, TRAILING:28, and MINLEN:20 (Bolger et al, 2014). The high-quality filtered reads were subsequently assembled using GetOrganelle v1.7.7.1 (Jin et al, 2020). The annotation was carried out using CPGAVAS2 (Shi et al, 2019), with the chloroplast genome of *Helicteres hirsuta* (accession number MW029459) used as the reference sequence. Challenges encountered during annotation, including ambiguous gene regions and uncertain gene boundaries, were resolved through manual curation using Unipro UGENE v45.1 (Okonechnikov et al, 2012) and NCBI Genomic Workbench v3.8.2 (Kuznetsov and Bollin, 2021). To ensure sequence completeness, the assembled chloroplast genome was verified to contain no ambiguous nucleotides (N) and to encode all 21 amino acids using Unipro UGENE v45.1. Genes lacking a start codon were manually corrected using the editing functions in NCBI Genomic Workbench v3.8.2. Finally, visualization of the circular chloroplast genome was generated using Organellar Genome DRAW (OGDRAW) via the MPI-MP Chlorobox platform (Greiner et al, 2019).

## Description of the chloroplast genome dataset

**Figure 1:**
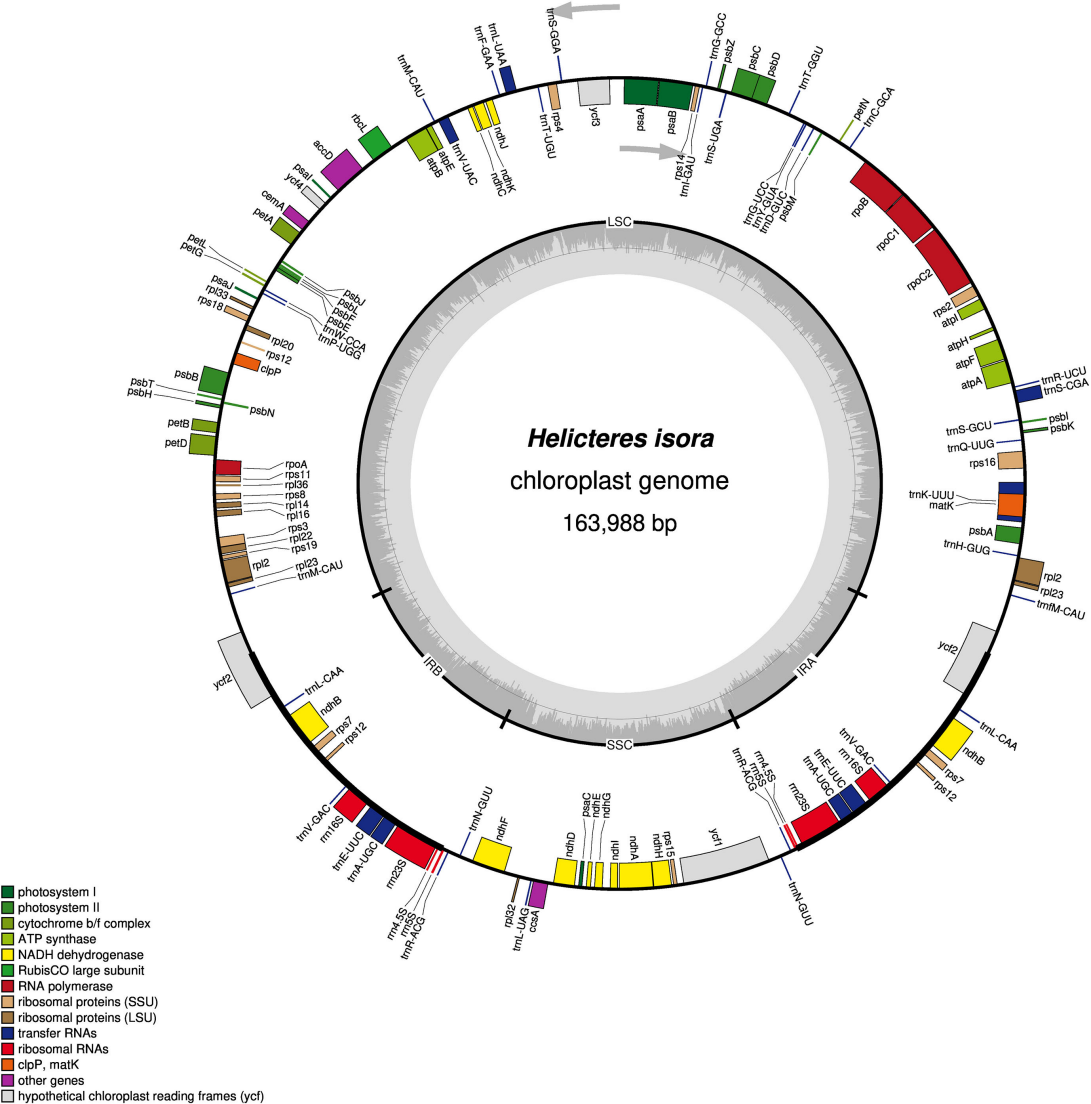
Circular map of the *Helicteres isora* chloroplast genome. Genes outside the circle are transcribed clockwise, while genes inside the circle are transcribed counterclockwise. Different colors indicate different functional groups. The inner gray circle represents GC content. LSC, large single-copy region; SSC, small single-copy region; IRa and IRb, inverted repeat regions.

The dataset comprises the complete chloroplast genome sequence of *Helicteres isora* assembled into a single circular molecule. The chloroplast genome has a total length of 163,988 bp and exhibits a quadripartite organization consisting of a large single-copy (LSC) region of 91,679 bp, a small single-copy (SSC) region of 19,789 bp, and two inverted repeat (IR) regions of 26,260 bp each (**Figure 1**). Base composition statistics indicate that adenine (31.10%) and thymine (32.20%) are more frequent than cytosine (18.60%) and guanine (18.00%), resulting in an overall GC content of 37.00%. Regional GC content values were calculated for the LSC (34.47%), SSC (37.78%), and IR (42.28%) regions to support comparative analyses of plastome composition. This GC enrichment in the IR regions is consistent with the presence of GC-rich rRNA genes and reflects a conserved compositional pattern commonly observed in chloroplast genomes of flowering plants (Lestari et al, 2024; Nugroho et al, 2025; Nuroniah et al, 2025).

A total of 113 unique genes were annotated in the chloroplast genome of *H. isora*, comprising 79 protein-coding genes (PCGs), 30 transfer RNA (tRNA) genes, and four ribosomal RNA (rRNA) genes (**Table 1**). These genes were classified into functional categories associated with photosynthesis, genetic self-replication, and other essential chloroplast functions. Genes involved in photosynthesis constitute a substantial proportion of the plastome and include those encoding subunits of photosystem I (*psa* genes), photosystem II (*psb* genes), the cytochrome b/f complex (*pet* genes), ATP synthase (*atp* genes), and NADH dehydrogenase (*ndh* genes), as well as the large subunit of Rubisco (*rbc*L). The presence and arrangement of these genes indicate a highly conserved photosynthetic apparatus in *H. isora*, consistent with other Malvaceae plastomes. Genes related to genetic self-replication include ribosomal protein genes of both the large (*rpl*) and small (*rps*) subunits, DNA-dependent RNA polymerase genes (*rpo*A–*rpo*C2), and a complete set of tRNA genes sufficient for chloroplast protein synthesis.

**Table 1:** List of genes in the *Helicteres isora* chloroplast genome.

Functional category	Group of gene	Gene
Self-replication	rRNA	*rrn*16S^d^, *rrn*23S^d^, *rrn*5S^d^, *rrn*4.5S^d^
	tRNA	*trn*A-UGC^d*^, *trn*C-GCA, *trn*D-GUC, *trn*E-UUC^d*^, *trn*F-GAA, *trn*G-GCC, *trn*G-UCC, *trn*H-GUG, *trn*I-GAU, *trn*K-UUU^*^, *trn*L-CAA^d^, *trn*L-UAA^*^, *trn*L-UAG, *trn*fM-CAU, *trn*M-CAU^d^, *trn*N-GUU^d^, *trn*P-UGG, *trn*Q-UUG, *trn*R-ACG^d^, *trn*R-UCU, *trn*S-CGA, *trn*S-GCU, *trn*S-GGA, *trn*S-UGA, *trn*T-GGU, *trn*T-UGU, *trn*V-GAC^d^, *trn*V-UAC^*^, *trn*W-CCA, *trn*Y-GUA
	Subunits of ATP synthase	*atp*A, *atp*B, *atp*E, *atp*F^*^, *atp*H, *atp*I
	Large subunit of ribosome	*rpl*14, *rpl*16, *rpl*2^d*^, *rpl*20, *rpl*22, *rpl*23^d^, *rpl*32, *rpl*33, *rpl*36
	Small subunit of ribosome	*rps*11, *rps*12^d**^, *rps*14, *rps*15, *rps*16^*^, *rps*18, *rps*19, *rps*2, *rps*3, *rps*4, *rps*7^d^, *rps*8
	Subunits of NADH-dehydrogenase	*ndh*A^*^, *ndh*B^d*^, *ndh*C, *ndh*D, *ndh*E, *ndh*F, *ndh*G, *ndh*H, *ndh*I, *ndh*J, *ndh*K
Photosyntesis	Subunits of cytochrome b/f complex	*pet*A, *pet*B, *pet*D^*^, *pet*G, *pet*L, *pet*N
	Subunits of photosystem I	*psa*A, *psa*B, *psa*C, *psa*I, *psa*J
	Subunits of photosystem II	*psb*A, *psb*B, *psb*C, *psb*D, *psb*E, *psb*F, *psb*I, *psb*J, *psb*K, *psb*L, *psb*M, *psb*N, *psb*T, *psb*Z, *ycf*3^**^
	Subunit of rubisco	*rbc*L
	DNA-dependent RNA polymerase	*rpo*A, *rpo*B, *rpo*C1^*^, *rpo*C2
	Subunit of Acetyl-CoA-carboxylase	*acc*D^**^
	c-type cytochrome synthesis gene	*ccs*A
Other function	Envelope membrane protein	*cem*A
	Protease	*clp*P
	Maturase	*mat*K
Conserved open reading frames	Conserved open reading frames	*ycf*1^**^, *ycf*2^d*^, *ycf*4

d, gene duplication; *, single intron; **, double intron.

Gene duplication associated with the inverted repeat regions was documented in the dataset. Several genes are present in two copies due to their localization within the IR regions, including the rRNA genes *rrn*16S, *rrn*23S, *rrn*5S, and *rrn*4.5S; the tRNA genes *trn*A-UGC, *trn*E-UUC, *trn*L-CAA, *trn*M-CAU, *trn*N-GUU, *trn*R-ACG, and *trn*V-GAC; and the protein-coding genes *ndh*B, *rpl*2, *rpl*23, *rps*7, *rps*12, and *ycf*2. In addition, intron-containing genes were identified and annotated. Single introns were recorded in genes such as *atp*F, *ndh*A, *ndh*B, *pet*D, *rpo*C1, and *rps*16, while genes including *ycf*1, *ycf*3, *rps*12, and *acc*D contain two introns. The *rps*12 gene exhibits a trans-splicing configuration, with exons distributed across different regions of the genome. Structural features of the *clp*P gene, including exon–intron organization, were also documented as part of the annotation output. All annotated genes, their functional categories, copy number, and intron status are summarized in **Table 1**. The complete chloroplast genome sequence and associated annotation files are publicly available through the NCBI GenBank database and are intended to support reuse in plastome annotation, molecular marker development, comparative genomics, and other data-driven studies involving medicinal plants.

## Data Availability

The dataset presented in this study is available in the NCBI GenBank database. The complete chloroplast genome sequence of Helicteres isora has been deposited under accession number PX570609.

## References

[B1] Alam A. Usman M. Parveen B. Ahmad S. (2024). “ Future directions and emerging trends in medicinal plant DNA barcoding,” in Ethnopharmacology and OMICS advances in medicinal plants, vol. 1. (Amsterdam, Netherlands: Elsevier), 219–237.

[B2] Andrews S. Krueger F. Segonds-Pichon A. Biggins L. Krueger C. Wingett S. (2010). FastQC: A quality control tool for high-throughput sequence data. Babraham Bioinf.

[B3] Bolger A. M. Lohse M. Usadel B. (2014). Trimmomatic: A flexible trimmer for Illumina sequence data. Bioinformatics 30, 2114–2120. doi: 10.1093/bioinformatics/btu170. PMID: 24695404 PMC4103590

[B4] Daniell H. Lin C.-S. Yu M. Chang W.-J. (2016). Chloroplast genomes: Diversity, evolution, and applications in genetic engineering. Genome Biol. 17, 134. doi: 10.1186/s13059-016-1004-2. PMID: 27339192 PMC4918201

[B5] Dayal R. Singh A. Ojha R. P. Mishra K. (2015). Possible therapeutic potential of Helicteres isora (L.) and its mechanism of action in diseases. J. Med Plants Stud. 3, 95–100.

[B6] Dobrogojski J. Adamiec M. Luciński R. (2020). The chloroplast genome: A review. Acta Physiol. Plant 42, 98. doi: 10.1007/s11738-020-03091-7. PMID: 30311153

[B7] Greiner S. Lehwark P. Bock R. (2019). OrganellarGenomeDRAW (OGDRAW) version 1.3.1: Expanded toolkit for the graphical visualization of organellar genomes. Nucleic Acids Res. 47, W59–W64. doi: 10.1093/nar/gkz238. PMID: 30949694 PMC6602502

[B8] Jin J. J. Yu W. B. Yang J. B. Song Y. dePamphilis C. W. Yi T. S. . (2020). GetOrganelle: A fast and versatile toolkit for accurate de novo assembly of organelle genomes. Genome Biol. 21, 241. doi: 10.1186/s13059-020-02154-5. PMID: 32912315 PMC7488116

[B9] Kanthale P. R. Biradar S. (2017). Pharmacognostic study of Helicteres isora L. Pharm. Biol. Eval 4, 47–51. doi: 10.26510/2394-0859.pbe.2017.07

[B10] Kumbhani N. R. Kuvad R. P. Thaker V. S. (2017). Development of linear model for leaf area measurement of two medicinally important plants: Helicteres isora L. and Vitex negundo L. Biol. Biotechnol. (India) 5, 57–60. doi: 10.7324/JABB.2017.50310

[B11] Kuznetsov A. Bollin C. J. (2021). “ NCBI Genome Workbench: Desktop software for comparative genomics, visualization, and GenBank data submission,” in Multiple sequence alignment: Methods and protocols. Ed. Katoh K. (New York, NY, United States: Humana Press), 261–295. doi: 10.1007/978-1-0716-1036-7_16 33289898

[B12] Lestari R. Magandhi M. Hariri M. R. Noviady I. Nugroho A. Indriani F. (2024). Characterization of the complete chloroplast genome of the endangered and endemic Bornean fruit Artocarpus tamaran Becc. Front. Plant Sci. 15, 1513364. doi: 10.3389/fpls.2024.1513364. PMID: 39726425 PMC11669497

[B13] Meena R. S. Das A. Yadav G. S. Lal R. (2018). Legumes for soil health and sustainable management (Singapore: Springer).

[B14] Nguyen H. D. Vu M. T. Do H. D. K. (2024). Characterization of the complete chloroplast genome of Helicteres hirsuta Lour. 1790 (Helicteriodeae: Malvaceae). Mitochondrial DNA Part B 9, 568–573. doi: 10.1080/23802359.2024.2345794. PMID: 38707209 PMC11067558

[B15] Nugroho A. Rustam E. Widyani N. Indriani F. Sudrajat D. J. Salim M. A. . (2025). Complete assembly of the organellar genome of Rubroshorea johorensis utilizing advanced long-read sequencing technologies. Front. Genet. 16, 1574266. doi: 10.3389/fgene.2025.1574266. PMID: 40438326 PMC12116502

[B16] Nuroniah H. S. Nugroho A. Indriani F. Suita E. Basyah B. Susanna S. . (2025). Chloroplast genome of Myristica fragrans: Assembly, annotation, and phylogenetic relationships. For. Sci. Technol. 21, 207–214. doi: 10.1080/21580103.2025.2482080. PMID: 37339054

[B18] Okonechnikov K. Golosova O. Fursov M. UGENE Team (2012). Unipro UGENE: A unified bioinformatics toolkit. Bioinformatics 28, 1166–1167. doi: 10.1093/bioinformatics/bts091. PMID: 22368248

[B17] Olejniczak S. A. Łojewska E. Kowalczyk T. Sakowicz T. (2016). Chloroplasts: State of research and practical applications of plastome sequencing. Planta 244, 517–527. doi: 10.1007/s00425-016-2531-7. PMID: 27259501 PMC4983300

[B19] Ray S. Satya P. Sharma L. Roy S. Bera A. Santra S. . (2022). “ Model plants in genomics,” in Plant genomics for sustainable agriculture (Singapore: Springer), 241–264.

[B20] Richards C. L. Alonso C. Becker C. Bossdorf O. Bucher E. Colomé-Tatché M. . (2017). Ecological plant epigenetics: Evidence from model and non-model species, and the way forward. Ecol. Lett. 20, 1576–1590. doi: 10.1111/ele.12858. PMID: 29027325

[B21] Shi L. Chen H. Jiang M. Wang L. Wu X. Huang L. . (2019). CPGAVAS2, an integrated plastome sequence annotator and analyzer. Nucleic Acids Res. 47, W65–W73. doi: 10.1093/nar/gkz345. PMID: 31066451 PMC6602467

[B22] Song B. H. Mitchell-Olds T. (2011). Evolutionary and ecological genomics of non-model plants. J. Syst. Evol. 49, 17–24. doi: 10.1111/j.1759-6831.2010.00107.x. PMID: 21394233 PMC3050529

